# The Stafford Interview: A comprehensive interview for mother-infant psychiatry

**DOI:** 10.1007/s00737-016-0683-8

**Published:** 2016-10-24

**Authors:** Ian Brockington, Prabha Chandra, Alessandra Bramante, Hettie Dubow, Walaa Fakher, LLuïsa Garcia-Esteve, Kristina Hofberg, Suaad Moussa, Bruma Palacios-Hernández, Ylva Parfitt, Pey-Ling Shieh

**Affiliations:** 1 University of Birmingham, Edgbaston, Birmingham, B15 2TT UK; 2 National Institute of Mental Health and Neurological Sciences, Bangalore, India; 3 Università di Torino, Turin, Italy; 4 Formerly Princess Margaret Hospital, Christchurch, New Zealand; 5 Cairo University, Cairo, Egypt; 6 Universitat Autònoma de Barcelona, Barcelona, Spain; 7 St George’s Hospital, Stafford, UK; 8 University of Sussex, Brighton, UK; 9 Chung Shan Medical University, Taichung, Taiwan

**Keywords:** Pregnancy, parturition, the puerperium, the mother-infant relationship

## Abstract

This article describes an interview exploring the social, psychological and psychiatric events in a single pregnancy and puerperium. It has been in development since 1992 and is now in its 6th edition. It takes approximately 2 h to administer and has 130 compulsory probes and 185 ratings. It is suitable for clinical practice, teaching and research.

## Introduction

All psychiatric specialities require a structured interview, thoroughly to explore their field of interest and practice. The Stafford Interview is an interview designed for all mental health specialties working in the mother-infant (perinatal) area. It explores the social, obstetric and psychological background, and psychiatric complications, of pregnancy, parturition and the puerperium.

Its scope is strictly limited to one episode of childbearing. The exploration of a single pregnancy, birth and postpartum period requires an interview that takes about 2 h to administer. Even if given in two sessions, this is at the limit of mothers’ tolerance. This interview, therefore, does not cover:

- Psychopathology in sufficient detail to meet the demands of operational criteria. There are several important symptom groups (anxiety, depression, psychosis, post-traumatic stress disorder), which have various defining criteria, whose inclusion would have distorted the interview and made it too long
- The personal history. To explore this with equal thoroughness, as in the Anne Roper Interview (Brockington 2014), takes several hours.

Its aims are to improve training and clinical practice and to provide a versatile research instrument. The Contextual Assessment of the Maternity Experience, which covers the same ground, has been used for impressive studies in normal mothers (Bernazzani et al. 2004, 2005), but has not, so far as we know, been published, or used in clinical work. If that is true, this is the only interview generally available to assess the mental health of expectant and newly delivered mothers and their interactions with the unborn and newborn child.

## Development

Work began, without funding, on the Queen Elizabeth Mother and Baby Unit, Birmingham, in 1993. With the collaboration of a Finnish group, led by Pirkko Niemela, and the participation of Mario Lanczik, Kristina Hofberg, Shoba George and trainees working on the unit, about 100 interviews were conducted with three editions, whose probes were published as an appendix to *Motherhood and Mental Health* (Brockington 1996). Other centres took an interest, and early editions were translated into German, Arabic, Kannada, Mandarin and Spanish. An early version was used in a study of mother-infant relationship difficulties (Loh and Vostanis 2004).

In 1998, a workshop was held in Birmingham, funded by the charity Women’s Mental Health (Chair Howard Voisey), leading to the development of a fourth, and shortly afterwards, a fifth edition, with 120 compulsory probes and 175 ratings. This has been translated into nine languages—Arabic, German, Italian, Japanese, Kannada, Maltese, Mandarin, Spanish and Polish. In 2006, the 5th edition was published, under the name of the Birmingham Interview for Maternal Mental Health, by Eyry Press (Brockington et al. 2006b). Only 35 exemplars of the book have been produced, but a copy is held by the British copyright libraries.

This edition was used in various centres for the following projects:

- In Birmingham and Christchurch, for the ‘Anglo-New Zealand study’ of over 200 mothers attending mother-infant services; this resulted in three publications—a validation of the Postpartum Bonding Questionnaire (Brockington et al. 2006c), a study of the definitions and frequency of severe mother-infant relationship disorders (Brockington et al. 2006a) and a study of anxiety, obsessions and morbid preoccupations in pregnancy and the puerperium (Brockington et al. 2006d)
- In Bangalore, a study of delusions in Indian mothers (Chandra et al. 2006); it has also been used extensively in training
- In Brighton, a Ph D thesis on post-traumatic stress disorder and parental anger (Parfitt 2012)
- In Milan, an Italian study of filicidal mothers (Bramante 2013)
- In Barcelona it has been used in training and clinical work, in a Ph D thesis (Palacios Hernández 2015), and the validation of a Spanish instrument (Torres et al. 2010)
- In Hong Kong, the validation of the Chinese version of the Postpartum Bonding Questionnaire (Siu et al. 2010)
- In Cairo, for a PhD thesis (Fakher 2015).

It has also been used in on-going research in Malta and Taiwan.

The time seemed ripe to assemble users from various nations, pool experience, and produce an improved version. A workshop, generously funded by St George’s Hospital, Stafford (Chief Executive Neil Carr), was held in September 2014, and resulted in the Stafford Interview, which is offered for general use.



 George's Hospital, Stafford in September 2014. They are (left to right) Ylva Parfitt (Brighton & Sweden), Prabha Chandra (Bangalore, India), Suaad Moussa (Cairo, Egypt), Ian Brockington (Bredenbury, UK), Alessandra Bramante (Milan, Italy), Hettie Dubow (Melbourne, Australia), Bruma Palacios-Hernández (Barcelona & Mexico), Lluisa Garcia-Esteve (Barcelona & Spain) and Kristina Hofberg (Stafford, UK)

## Description

### Principles

The interview can be conducted in the community or in the clinical setting. It is designed for use worldwide, with mothers of many cultural backgrounds. The interviewer can omit probes that are culturally inappropriate.

The word ‘mother’ refers to the subject or patient throughout. The whole interview can be conducted up to 1 year after childbirth or during pregnancy and the postpartum period in separate prepartum and postpartum sections. It can be conducted in a single setting but (because of its length) more conveniently in two or three sessions.

Unless an audio- or videotape record is kept, the interviewer is instructed to record in narrative the evidence for each rating, so that other raters, not present at the interview, can judge the nature and severity of symptoms or items present; so far as possible, the interviewer should write down the subject’s own words, and there is sufficient space in the schedule for this purpose. The verbatim record allows other ratings to be made, not in the schedule. It allows pairs of raters to rate independently, measure their reliability and, after discussion, agree consensus ratings (Brockington et al. 1992); we believe that this is the correct way to establish reliability, not by borrowing ‘reliability’ from co-trained experts, working under ideal conditions and knowing that their accuracy is under scrutiny (Reid 1970; Taplin and Reid 1973). The verbatim record also allows ratings to be compared between subjects (cross-sectional analysis); in a large sample of mothers, all those with a particular rating can be compared, and adjustments made.

Since the interview makes no attempt comprehensively to explore psychopathology, those who need to focus on one disorder (such as depression) can add additional questions or self-rating scales. Recommendations for add-on scales or interview questions are made at appropriate points.

Compulsory probes are printed in bold type, additional questions in regular type and instructions to interviewers in italics. ‘If’ clauses are provided to shorten the interview.

Coding instructions are given on the page opposite the questions. A rating of ‘8’ indicates that the rating is not applicable to this mother, and ‘9’ that the rating cannot be made, because of inadequate information.

During the interview the mother will, to some extent, follow her own lines of thought, and it will be necessary to move from one section to another. To facilitate this movement, sections are printed on paper of different colours, and recommendations are offered for a simple method of binding.

### Contents

The prepartum section is in four parts—introduction (white paper), the social, psychological and obstetric background to pregnancy (green paper), the unborn child (yellow paper) and prepartum emotional changes and psychiatric disorders (blue paper).

The interview begins with general enquiries about the circumstances of referral or selection, the most important events in the mother’s life, her psychiatric and obstetric history and the people who will be important to this baby.

The section headed ‘social, psychological and obstetric background’ has the following subsections, with exploratory questions on

- The mother’s circumstances at the time of conception, planning of pregnancy, and her response (and the response of others), to the diagnosis and announcement of pregnancy
- Each trimester, covering adjustment to pregnancy, physical health, insomnia and emotional response to pregnant appearance
- Relationships with the husband, partner or (if different) father of the baby, family of origin, family by marriage, older or step children, friends and confidantes, and the available emotional and practical support
- Changes in life style, sacrifices made, adverse events and hardship, with an overall assessment of the favourable and adverse factors.

Attention is directed to the unborn child—any social and medical concerns, and (if culturally appropriate) interaction with the foetus.

The section on prepartum psychiatric disorders begins with a question about positive mental health and an overview of psychiatric symptoms. The mother’s worries and concerns are rated under nine categories, for example, the fear of parturition. General probes, without a full inventory of symptoms, are used to explore anxiety, obsessive/compulsive symptoms, irritability, depression and other psychiatric disorders, including psychosis. Here, the interview can be augmented by additional schedules or self-rating scales. General ratings are made on onset and duration, treatment and role impairment.

The postpartum section is in five parts—parturition (orange paper), the social, psychological and medical background to the puerperium (green paper), postpartum psychiatric disorders (blue paper), the mother infant relationship (yellow paper) and observations, summary, diagnosis and treatment plan (white paper).

The exploration of parturition covers the obstetric events, mental state during and after labour and condition of the baby at its birth.

The social, psychological and medical background to the puerperium begins with probes about a range of postpartum events such as the mother’s reaction to the newborn, breastfeeding and sleep deprivation. The relationship with the father of the baby and family members is dealt with in the same way as during pregnancy, with the addition of questions that relate to their attitudes to the baby. Cultural issues such as postpartum and pregnancy rituals, and concern about the gender of the infant, have been included.

As in pregnancy, the exploration of postpartum psychiatric disorders begins with a question about positive mental health and an overview of psychiatric symptoms. The mother’s worries and concerns are rated with a different set of ten categories, for example, the pathological fear of cot death. General probes are used to explore anxiety, obsessive/compulsive symptoms, irritability, depression and other psychiatric disorders. Two additional anxiety ratings are concerned with phobic avoidance of the baby and intrusive behaviour. In depressed mothers, there are additional questions with her maternal role and the involvement of the infant in suicidal ideas. A page is devoted to postpartum psychosis, with a caveat that most of the evidence will come from observations about the mother’s behaviour. There are general ratings of postpartum psychiatric disorder, similar to those in pregnancy.

The section on the mother-infant relationship has three pages of probes and ratings; it can be used as a self-standing instrument, together with a self-rating scale such as the PBQ (Brockington et al. 2006c). These pages deal with

- Infant characteristics and maternal involvement in care
- The mother’s emotional response to her infant
- Anger and abuse.

The interview concludes with questions about satisfaction with psychiatric treatment and the desire for further children. The interviewer records impressions, including abnormal behaviour. Consideration is given to any particular risks to this mother or the child. On the final page, there is a summary of the main features, and a diagnosis and treatment plan.

Table 1 details the probes and ratings. In total, there are 130 probes and 185 ratings—about ten more than in the 5th edition of the Birmingham Interview.

**Table 1:** Probes and ratings

	Section	Sub-section(s)	Compulsory probes	Ratings
Prepartum interview	Introduction	Circumstances of referral	8 plus enquiries into psychiatric and obstetric history	None
		Personal and psychiatric history		
		Obstetric and gynaecological history		
	Social, psychological and obstetric background to pregnancy	Circumstances at conception	7	8
		1st, 2nd and 3rd trimesters	13	7
		Relationship with baby’s father, families and significant others	12	11
		Changes in life style	3	5
		Positive and negative scores	Review of the above	2
	Wellbeing of unborn child		6	7
	Prepartum psychiatric disorders	Positive mental health	1	1
		General account of mental disorder	1	None
		Worrying and morbid preoccupations	1	9
		Anxiety	1	1
		Obsessive/compulsive disorder	2	2
		Irritability	1	3
		Depression	2	3
		Other psychiatric disorders	3	5
		General ratings	Review of the above	1 and a chart
		Treatment	Conditional question	2
		Effect on role performance	3	3
Postpartum interview	Parturition	Course of obstetric events	7	7
		Mental state during and after labour	3	4
		The newborn	5	7
	Social, psychological and obstetric background to the puerperium	Postpartum events	17	19
		Relationship with baby’s father, families and significant others	11	14
		Positive and negative scores	Review of the above	2
	Postpartum psychiatric disorders	Positive mental health	1	2
		General account of mental disorder	1	
		Worrying and morbid preoccupations	4	10
		Anxiety	1	3
		Obsessive/compulsive disorder	2	3
		Irritability	1	3
		Depression	2	4
		Psychosis	None	4
		Other psychiatric disorders	5	4
	The mother-infant relationship	Infant characteristics and maternal involvement in care	10	6
		The mother’s emotional response to her infant	4	6
		Anger and abuse	3	6
	Postpartum psychiatric disorders	General ratings	Review of the above	1 and a chart
		Treatment	Conditional question	2
		Effect on role performance	4	4
	Conclusion		5	2
Totals			130	185

## Deployment of the interview

### Uses

The interview can be used for training, clinical practice and research. Its value in training has been exploited in Bangalore and Barcelona, where all trainees conduct interviews. Even a single interview can foster awareness of the wide scope of mother-infant psychiatry and demonstrate the tactful exploration of all areas.

In clinical practice, a 2-h interview is well tolerated by mothers, who appreciate the thorough exploration of their problems. Mothers are able to talk openly, freely in a relaxed way about sensitive issues like foetal abuse, child maltreatment, filicidal ideas, intimate partner violence and suicide. It is often a relief to speak in detail about their suffering, and find understanding of areas, which, in oppressive cultures, arouse obloquy, shame and guilt. The interviews are often positive and therapeutic in themselves. They allow complex clinical presentations to be broken down to their specific components, each with an optimal treatment. The interview provides an in-depth account of the transition to parenthood and pays attention to all areas of the childbearing process, including some, such as the planning of and acceptance of pregnancy, parturition, and the mother-infant relationship, which are rarely explored by mental health professionals.

In research, the Birmingham Interview has already been used for the validation of self-report questionnaires, and the Stafford Interview can play a similar role with other tools and ‘instruments’. In the Anglo-New Zealand study, it contributed to the cross-cultural comparison of childbearing disorders: for example, it showed that emotional rejection of the child was encountered not only in Caucasian and *Pakeha* mothers but in Pakistani and Sikh mothers in Birmingham and the Maori in Christchurch. Comparisons between Indian, African and South or Central American centres would be of great interest.

With its self-standing prepartum and postpartum stages, it is ideal for the cohort studies that are so badly needed to determine the predictors of postpartum disorders and provide a platform for prevention.

There are plans to hold a conference in Stafford in May 2018 to review research conducted with the Stafford Interview and give preliminary thoughts to improvements.

## Availability

The Stafford Interview is safeguarded by copyright—with Ian Brockington for the English version and with the translators for other versions. It is available free of charge. The English language version has already been advertised on the World Wide Web (<http://staffordinterview.sssft.nhs.uk>) and can be obtained by e-mail request. Once it is known who is using it and for what purpose, it will be sent by e-mail attachment, with recommendations and instructions for printing and binding.

The Arabic version can be obtained from Suaad Moussa, suaadmoussa@yahoo.com, the Italian version from Alessandra Bramante, a.bramante@libero.it, the Kannada and Hindi versions from Prabha Chandra, prabhasch@gmail.com, the Mandarin version from Peyling Shieh, peylings@gmail.com, the Spanish version from LLuïsa Garcia-Esteve, lluisagarcia@gmail.com, and the Turkish version from Gökşen Yukşel, drgoksenyuksel@gmail.com.
